# TTV Viremia and Immune Responses Following Vaccination Against Mpox and Dengue Viruses

**DOI:** 10.3390/vaccines14050441

**Published:** 2026-05-15

**Authors:** Claudia Minosse, Pietro Giorgio Spezia, Sara Belladonna, Aurora Bettini, Giulia Matusali, Francesca Colavita, Stefania Notari, Linda Petrone, Marta Tiberi, Alessandro Rosario Cavasio, Valentina Mazzotta, Luigi Rosa, Eleonora Cimini, Daniele Focosi, Delia Goletti, Emanuele Nicastri, Andrea Antinori, Fabrizio Maggi

**Affiliations:** 1 Laboratory of Virology and Laboratories of Biosecurity, National Institute for Infectious Diseases Lazzaro Spallanzani-IRCCS, 00149 Rome, Italy; 2 Cellular Immunology and Pharmacology Laboratory, National Institute for Infectious Diseases Lazzaro Spallanzani-IRCCS, 00149 Rome, Italy; 3 Translational Research Unit, National Institute for Infectious Diseases Lazzaro Spallanzani-IRCCS, 00149 Rome, Italy; 4 Clinical and Research Department, National Institute for Infectious Diseases Lazzaro Spallanzani-IRCCS, 00149 Rome, Italy; 5 North-Western Tuscany Blood Bank, Pisa University Hospital, 56126 Pisa, Italy

**Keywords:** TTV, MPXV, DGV, MVA-BN, TAK-003, Immune responses

## Abstract

**Background**: Torquetenovirus (TTV) viremia is increasingly recognized as a biomarker of host immune competence. We assessed the association between baseline TTV DNA levels and immune responses to the Mpox virus (MPXV) and dengue virus (DGV) vaccines in two prospective cohorts. **Methods**: A total of 248 individuals were enrolled, and TTV DNA was quantified before vaccination. Humoral and cellular responses to MVA-BN (for MPXV) and QDENGA (for DGV) vaccines were measured by using serology, neutralization assays, and interferon-γ ELISpot, and correlations with TTV viremia were investigated. **Results**: TTV DNA was detected in 81.2% of individuals, with a significantly higher prevalence and viral loads in the Mpox-Vac group than in the DGV-Vac group. Between both groups, the only significant association observed was an inverse correlation between pre-vaccination TTV load and DGV neutralizing antibody titers in the DGV-Vac group and was limited to the subset of TTV-positive individuals; no additional correlations with antibody and T responses were identified. For the Mpox-Vac group, stratified analyses in people living with HIV (PLWH) confirmed this lack of association. **Conclusions**: TTV viremia does not predict vaccine immunogenicity in immunocompetent or mildly immunosuppressed individuals. These results, which derive from within-cohort analyses and do not rely on direct comparisons between heterogeneous vaccine populations, support the role of TTV as a marker of immune status along a continuum of immunosuppression, with predictive value likely confined to populations with more severe immune impairment.

## 1. Introduction

Torquetenovirus (TTV) is a small, non-enveloped, circular, single-stranded DNA virus belonging to the family Anelloviridae. It is highly prevalent in the human population, with detection rates exceeding 70–90% in healthy adult populations [1]. Despite its extraordinary ubiquity, TTV has not been associated with any specific clinical disease. Rather than acting as a classical pathogen, TTV is increasingly regarded as a component of the human virome whose replication is tightly regulated by host immune surveillance mechanisms [2,3]. In immunocompetent individuals, viral replication is typically maintained at relatively stable and moderate levels. In contrast, in conditions of impaired immune function, such as solid organ transplantation, hematological malignancies, primary immunodeficiencies, or uncontrolled HIV infection, TTV viral load (VL) increases significantly.

These observations have led to the recognition of TTV viremia as a quantitative surrogate marker of immunosuppression [4,5,6]. Over the past decade, TTV quantification has been proposed as a tool to guide immunosuppressive therapy in transplant recipients, where excessive immunosuppression correlates with high TTV VL and increased infection risk, whereas insufficient immunosuppression is associated with low TTV levels and risk of rejection. Beyond transplantation, TTV has also been investigated as a biomarker of immune function in oncology, chronic viral infections such as HIV, and in the context of immune reconstitution following antiviral therapy or severe systemic infections, including COVID-19. These findings collectively support the concept that TTV replication mirrors the net state of immune suppression along a continuous spectrum [7,8,9,10,11]. Interestingly, high TTV VLs have been associated with a poor response to vaccination in people with severe immunosuppression such as transplant patients, and this finding has led to the hypothesis that the virus could potentially serve as a predictive biomarker of vaccine-induced immunity in other types of populations [12,13,14,15]. However, the effectiveness of TTV as a predictive biomarker for immune responses to vaccination in non-transplant populations has been so far poorly investigated. This point is particularly relevant also considering the recent authorization and implementation of new vaccines targeting emerging infections, such as the Modified Vaccinia Ankara–Bavarian Nordic (MVA-BN) vaccine for Mpox virus (MPXV) and the tetravalent dengue vaccine TAK-003 (Qdenga^®^, Takeda, Osaka, Japan) for dengue virus (DGV). These vaccines are increasingly administered to specific target populations, including immunocompromised individuals, such as people living with HIV (PLWH) and those with pre-existing comorbidities, as well as immunocompetent subjects [16,17,18].

In this study, we evaluated the association between baseline TTV viremia and the magnitude of vaccine-induced immune responses to MVA-BN and TAK-003 vaccines. Specifically, we examined correlations between pre-vaccination TTV VL and post-vaccination IgG titers, neutralizing antibodies, and interferon-γ-producing T cell responses. The primary objective was to determine whether TTV VL could serve as a predictive biomarker of vaccine immunogenicity in immunocompetent or mildly immunosuppressed individuals. A secondary objective was to explore whether TTV may function as a stratification tool across varying degrees of immune competence.

## 2. Materials and Methods

### 2.1. Study Population and Samples

Subjects were enrolled in the vaccination campaigns against MPXV and DGV launched by the National Institute of Infectious Diseases Lazzaro Spallanzani-IRCCS on 8 August 2022 and on 23 February 2024, respectively. Participants were enrolled under two observational protocols (Mpox-Vac and DGV-Vac groups). For the Mpox-Vac group blood samples were collected before receiving vaccination (T1) and one month after the last dose (T2 and T3, respectively); for the DGV-Vac group blood samples were collected prior to the administration of the first TAK-003 vaccine dose (T1) and three months later (T3), immediately before the administration of the second dose.

All participants provided written informed consent. Both protocols were approved by the INMI Lazzaro Spallanzani/Lazio Area 4 Ethical Committee with approval number 41z, 2022, and 96-2023, respectively.

### 2.2. TTV DNA Quantification

The TTV VL was measured in the serum collected at T0 by real-time PCR performed on the fully automated Panther Fusion^®^ molecular system (Hologic, Inc., Marlborough, MA, USA), as previously described [19]. Briefly, 250 µL of the sample was extracted by using the Panther Fusion^®^ Extraction Reagent-S^®^ (Hologic, Inc., Marlborough, MA, USA), and 5 µL of eluate was used in 25 µL RT-PCR reactions in the Open Access RNA/DNA Enzyme Cartridge^®^ (Hologic, Inc., Marlborough, MA, USA). The final concentrations of the PCR reaction components are as follows: KCl 50 mM; MgCl2 4 mM; Tris (pH 8.0) 10 mM; Forward Primer (AMTS 5′-GTGCCGIAGGTGAGTTTA-3′, position nucleotides 177 to 194 according to isolate NCBI Reference Sequence NC_002076) 1 µM; Reverse Primer (AMTAS 5′-AGCCCGGCCAGTCC-3′, position nucleotides 226 to 239) 1 µM; Probe (AMTPTU 5′-TCAAGGGGCAATTCGGGCT-3′, position nucleotides 205 to 223) 0.3 µM; Internal Control Primer^®^ (Hologic, Inc., Marlborough, MA, USA) 0.6 µM; Internal Control Probe^®^ (Hologic, Inc., Marlborough, MA, USA) 0.6 µM.

The primers and probe target a highly conserved region of the TTV genome and are part of the most widely used and clinically validated PCR assay for TTV quantification [20]. Thermal cycling conditions were as follows: 2 min to 95 °C, followed by 45 cycles of 8 s at 95 °C and 28 s at 55 °C. The limit of detection (LOD) of the Panther Fusion^®^ TTV real-time PCR method was 42 copies/mL (1.6 log copies/mL). The results are expressed in log copies/mL. A value of TTV < 1.6 log copies/mL was assigned to undetectable TTV VL.

### 2.3. Humoral Immune Response Assessment

MPXV- and DGV-specific IgG antibodies and neutralizing antibodies (nAbs) were assessed using virus-specific methodologies.

MPXV-specific IgGs were measured, as previously described [17,21], by an in-house indirect immunofluorescence assay (IFA) using slides prepared with Vero E6 cells (ATCC) infected with an MPXV isolate obtained from a patient’s skin lesion (GenBank: ON745215.1); sera were tested in serial two-fold dilutions starting from 1:20. In contrast, DGV-specific IgGs (serotypes 1–4) were quantified using a commercial ELISA kit (EUROIMMUN, Lübeck, Germany) processed on the EUROIMMUN Analyzer I-2P (EUROIMMUN, Lübeck, Germany), according to the manufacturer’s instructions; IgG titers > 22 relative units (RUs) were considered positive, 16–22 RU equivocal, and <16 RU negative, with equivocal samples retested and classified as positive if persistent. Functional neutralizing activity was subsequently evaluated for both viruses using cell-based assays, as previously described [21].

MPXV nAbs were measured by a 50% plaque reduction neutralization test (PRNT_50_), in which heat-inactivated sera (30 min at 56 °C), starting from a 1:10 dilution, were incubated with 100 TCID_50_ of MPXV prior to infection of Vero E6 cells, and neutralization titers were defined as the serum dilution reducing plaque numbers by ≥50% compared with virus controls after 5 days of incubation. DGV nAbs were assessed using a virus neutralization test (VNT) against DGV2 (New Guinea C strain; NCPV 0006041v) as follows: heat-inactivated sera (starting dilution 1:10) were serially titrated, incubated with 100 TCID_50_ of virus, and added to Vero E6 cell monolayers, with neutralization defined as the highest serum dilution preventing virus-induced cytopathic effect at day 6 post-infection [22]. For both neutralization assays, titers ≥ 1:10 were considered positive, and appropriate positive and negative controls were included.

### 2.4. Peripheral Blood Mononuclear Cell (PBMC) Isolation and T Cell Stimuli

Peripheral blood mononuclear cells (PBMCs) were isolated from peripheral blood samples collected in heparinized tubes using density gradient centrifugation with Ficoll-Paque Premium (GE Healthcare Life Sciences, Chicago, IL, USA). The isolated PBMCs were cryopreserved in fetal bovine serum (FBS; Gibco, Life Technologies, Carlsbad, CA, USA) supplemented with 10% dimethyl sulfoxide (DMSO; Gibco, Life Technologies, Carlsbad, CA, USA) and stored in liquid nitrogen until further use.

In this study, the analysis of the T cell response to Mpox vaccination was performed with the MVA-BN vaccine suspension [JYNNEOS (Smallpox and Monkeypox Vaccine, Live, non-replicating)] [17].

Concerning DGV T cell stimuli, MegaPools (MPs) of peptides derived from the sequential lyophilization of large numbers of peptides were used, and were therefore useful in various assays for measuring T cell responses [23]. For measuring the DGV-specific T cell response the DGV CD4 MP and DGV CD8 MP were used. These peptide pools were designed based on experimentally derived T cell epitopes identified following infection with all four DGV serotypes. Briefly, the DGV CD4 MP consists of 180 15-mers peptides, whereas the DGV CD8 MP includes 268 9/10-mers peptides [24,25,26,27,28,29,30]. These peptide pools were pooled, lyophilized, reconstituted in DMSO, aliquoted, and stored at −20 °C until use.

### 2.5. IFN-γ -ELISPOT

Interferon-γ-producing specific T cells to the MVA-BN vaccine were assessed by ELISPOT assay, as previously described [17]. PBMC were thawed and suspended in complete medium [RPMI-1640 added of 10% fetal bovine serum, 1% L-glutamine, and 1% penicillin/streptomycin (Euroclone, Milan, Italy)]. Live PBMCs were counted by trypan blue exclusion and plated at 3 × 10^5^ cells/well in ELISPOT plates (Human IFN-y ELISpot plus kit; Mabtech, Nacka Strand, Sweden). MVA-BN vaccine suspension (MOI 1) was used for simulating cells. Co-stimuli αCD28/αCD49d (1 µg/mL, BD Biosciences, Milpitas, CA, USA) and used for 20 h stimulation at 37 °C (5% CO_2_).

After incubation, the ELISPOT assay was performed according to the manufacturer’s instructions. The spontaneous IFN-γ release was calculated in unstimulated culture (background), and a superantigen (SEB, 200 nM, Sigma-Aldrich/Merck, Darmstadt, Germany) was used as a positive control. Results are expressed as spot-forming cells per 10^6^ PBMCs (SFC/10^6^ PBMCs) in stimulated cultures after subtracting background (unstimulated culture).

For the DGV IFN-γ -ELISPOT, thawed PBMCs were stimulated overnight with DENV CD4 MP and DGV1-4 CD8 MP at 1 µg/mL. Cells left unstimulated were included as a negative control, PBMCs stimulated with staphylococcal enterotoxin B (SEB, Sigma-Aldrich/Merck, Darmstadt, Germany) at 200 ng/mL as positive control and cells stimulated with a SARS-CoV-2 Spike-peptide pool (Miltenyi PepTivator^®^SARS-CoV-2 Prot_S1, Prot S, and Prot S+, Miltenyi Biotec, Bergisch Gladbach, Germany) at 0.1 μg/mL were included to evaluate the T cell specific-response to an unrelated antigen. The IFN-γ-ELISPOT assay was performed according to the manufacturer’s instructions. The criteria used to define a positive responses were: (1) more than five spot-forming cells (SFCs) per well in stimulated conditions after subtraction of the negative control, and (2) a >2 stimulation index (SI: number of SFCs in the stimulated wells divided by the number of SFCs in the negative control) [30,31].

### 2.6. Statistical Analysis

Categorical data were presented as frequencies and percentages. Continuous variables were presented as median and inter-quartile range (25th percentile; 75th percentile). Continuous variables were compared via the non-parametric Mann–Whitney U test. Spearman’s rank correlation was employed to assess relationships between continuous variables. All statistical tests were two-tailed, with a significance threshold of *p* < 0.05. Analyses were conducted using Prism v.8.0.2 software. Correlation analyses were conducted as exploratory assessments across distinct immune parameters and cohorts and were not intended as a single confirmatory multiple-testing framework. Undetectable TTV values were assigned the limit of detection (1.6 log copies/mL) and treated as left-censored. Given the use of non-parametric rank correlations, this approach is unlikely to have substantially influenced the results.

## 3. Results

### 3.1. Study Population

A total of 248 participants were enrolled within the following two observational protocols:

(a) Mpox-Vac group: A total of 164 individuals enrolled in the prospective observational study on the safety, immunogenicity, and acceptability of the MVA-BN (JYNNEOS) vaccine in at-risk populations. Eighty-nine (54.3%) had a history of smallpox vaccination and received a single vaccine dose; 75 (45.7%) were vaccine-naïve and received two doses. All were male, with 90.2% identifying as men who have sex with men (MSM). Eighty-nine subjects were people living without HIV (PLWoH) and 75 (45.7%) were PLWH, all on highly active antiretroviral therapy (HAART). Among the 75 PLWH, 77.3% had a CD4+ T cell count > 500 cells/μL, 18.7% had counts between 200 and 500 cells/μL, and 4% had counts < 200 cells/μL. The overall median age was 49 years (IQR 41–55); specifically, 53 years (IQR 50–57) in the vaccine-experienced group and 40 years (IQR 34–45) in the vaccine-naïve group (*p* < 0.0001). No other characteristics showed significant differences between the two groups. (b) DGV-Vac group: A total of 84 individuals enrolled in the observational study on immune response to the TAK-003 (Qdenga^®^, Takeda, Osaka, Japan) DGV vaccine. Forty-two (50%) were individuals with no prior DGV infection, while the remaining 42 had a history of DGV infection. Both groups received the first dose of the TAK-003 vaccine. Forty (48.78%) were males; the median age was 42 years old (IQR = 31.0–63.25 years). Specifically, in DGV-naïve group, 24 were male (57.14%) with the median age of 43.5 years old (IQR = 30.25–64.75 years); in contrast, in the group of individuals with a history of DGV infection, 16 (38.1%) were male with the median age of 42 years old (IQR = 32.75–59.25 years).

### 3.2. TTV Results

**Figure 1:**
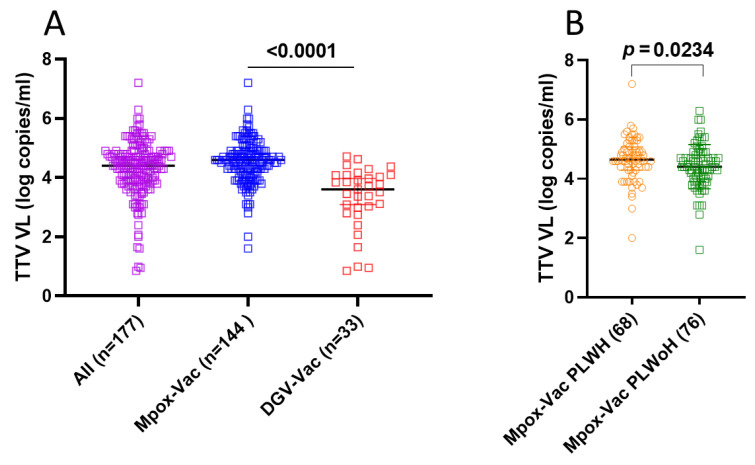
(**A**) Pre-vaccination TTV VL in the Mpox-Vac group (blue), DGV-Vac group (red) and overall patients (purple). Median, upper, and lower quartiles are indicated by horizontal lines. (**B**) Pre-vaccination TTV VL in PLWH (orange) and PLWoH (green) within the Mpox-Vac group. Median, upper, and lower quartiles are indicated by horizontal lines. Footnotes: TTV: Torquetenovirus, VL: viral load.

TTV prevalence and VL were assessed in serum samples collected at T1. Overall, 177 out of 248 samples (71.4%) were positive for TTV DNA, with a median VL of 4.4 (IQR 3.9–4.8) log copies/mL. Specifically, 144 out of 164 samples (87.8%) and 33 out of 84 samples (39.3%) resulted TTV DNA-positive in the Mpox-Vac group [median TTV VL: 4.6 (IQR 4.1–4.9) log copies/mL] and the DGV-Vac group [median TTV VL: 3.6 (IQR 2.9–4.1) log copies/mL], respectively (Figure 1A). There was a highly significant difference between TTV VL assessed in the Mpox-Vac group versus the DGV-Vac group (*p* < 0.0001). These inter-group differences are presented for descriptive purposes only and are not intended to support causal inferences, given the intrinsic demographic and clinical heterogeneity of the cohorts.

In the Mpox-Vac group, there was a statistically significant difference (*p* = 0.023) between TTV VL in 75 PLWH (median TTV VL: 4.6; IQR 4.3–5.1 log copies/mL) and 89 PLWoH (median TTV VL: 4.4; IQR 4.0–4.8 log copies/mL) (Figure 1B).

**Figure 2:**
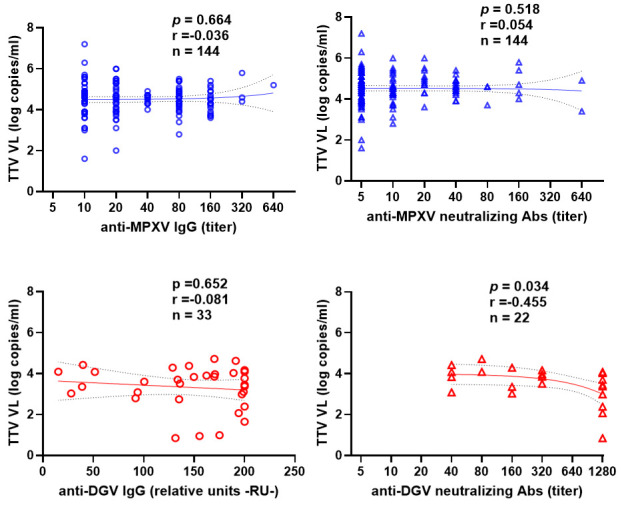
Correlation between pre-vaccination TTV VL and anti-MPXV or anti-DGV IgGs (circle) and nAbs (triangle) titers after first vaccine dose in the Mpox-Vac group (blue), and the DGV-Vac group (red). The best-fit line and 95% confidence bands of the linear regression analysis are represented by solid and dotted lines, respectively. Footnotes: TTV: Torquetenovirus, VL: viral load, MPXV: Mpox virus, DGV: Dengue virus.

We analyzed the correlation between baseline TTV VL and the titers of anti-MPXV/anti-DGV IgG and neutralizing Ab following the first dose. As shown in Figure 2, no significant correlation was found between pre-vaccination TTV VL and serum IgG levels for either the Mpox-Vac group (r = −0.036, *p* = 0.664; blue circles) or the DGV-Vac group (r = −0.081, *p* = 0.652; red circles).

Similarly, no significant correlation was observed between pre-vaccination TTV VL and anti-MPXV neutralizing Abs titers (r = 0.054 and *p* = 0.518, blue triangles). Neutralizing antibody analyses in the DGV-Vac cohort were limited to DENV-2 and should then be interpreted as a partial assessment of tetravalent vaccine immunogenicity. However, a correlation was observed between pre-vaccination TTV VL and anti-DGV nAbs titers in the DGV-Vac group (r = −0.455, *p* = 0.034, red triangles). This analysis was restricted to TTV-positive individuals within the DGV-Vac cohort (n. 33), and the observed association should therefore be interpreted as exploratory.

**Figure 3:**
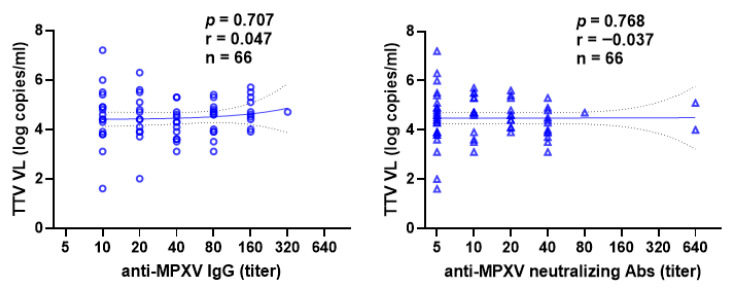
Correlation between pre-vaccination TTV VL and anti-MPXV IgGs (circle) and nAbs (triangle) titers after second vaccine dose in the Mpox-Vac group. The best-fit line and 95% confidence bands of the linear regression analysis are represented by solid and dotted lines, respectively. Footnotes: TTV: Torquetenovirus, VL: viral load, MPXV: Mpox virus.

In 75 smallpox vaccine-naïve or smallpox non-primed subjects who received a second dose of Mpox vaccine, the correlation between pre-vaccination TTV VL and anti- MPXV IgG and nAb titers after the second dose was also evaluated. Even in this case, no correlation was observed (Figure 3).

**Figure 4:**
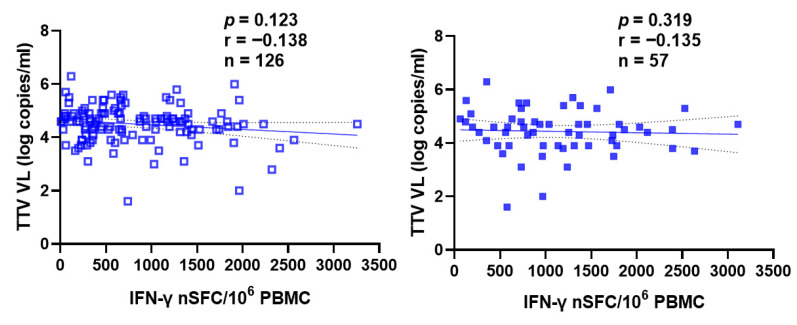
Correlation between pre-vaccination TTV VL and T cell-specific responses to the MVA-BN vaccine (assessed by interferon-γ ELISPOT) after first (empty square) or second (full square) vaccine dose. Results are expressed as number of spot-forming cells per 10^6^ PBMCs in stimulating cultures. The best-fit line and 95% confidence bands of the linear regression analysis are represented by solid and dotted lines, respectively. Footnotes: TTV: Torquetenovirus, VL: viral load; PBMC: peripheral blood mononuclear cell.

**Figure 5:**
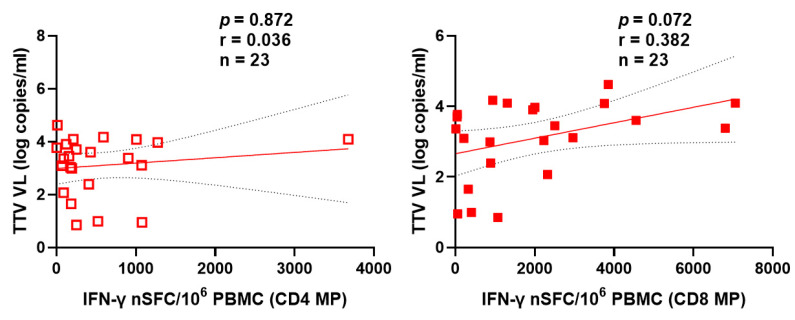
Correlation between pre-vaccination TTV VL and IFN-γ-producing T cells specific for DGV CD4 (empty square) and CD8 MPs (full square) detected by ELISPOT after TAK-003 (Qdenga^®^) vaccination. The T-cell response is expressed as SFC per 10^6^ PBMCs. The best-fit line and 95% confidence bands of the linear regression analysis are represented by solid and dotted lines, respectively. Footnotes: TTV: Torquetenovirus, VL: viral load; PBMC: peripheral blood mononuclear cell, CD: Cluster Definition; MP: MegaPool.

Figure 4 and Figure 5 also show the absence of correlation between pre-vaccine TTV VL and specific T cell responses to the MVA-BN and TAK-003 (Qdenga^®^) DGV vaccine, assessed by interferon-γ ELISPOT after the first or second dose of vaccine in the case of Mpox (Figure 4) and specific for helper T lymphocytes (CD4+) and cytotoxic (CD8+) in the case of DGV (Figure 5).

**Figure 6:**
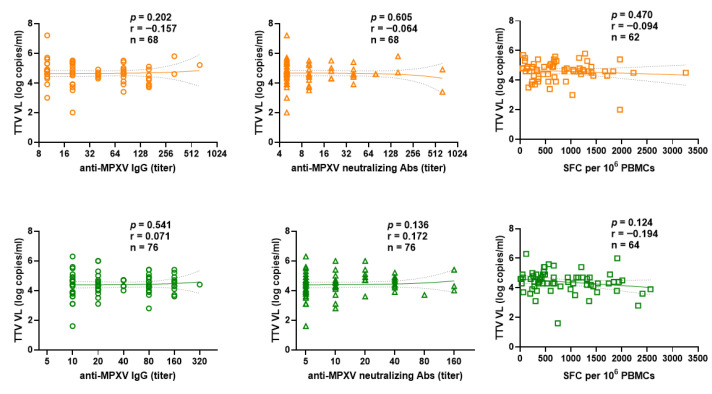
Correlation between pre-vaccination TTV VL and anti- MPXV IgGs (circle), nAbs (triangle) titers and T cell-specific responses to the MVA-BN vaccine (assessed by interferon-γ ELISpot) (square) after first vaccine dose in PLWH (orange) and PLWoH (green) within the Mpox-Vac group. The best-fit line and 95% confidence bands of the linear regression analysis are represented by solid and dotted lines, respectively. Footnotes: TTV: Torquetenovirus, VL: viral load; MPXV: Mpox virus, PBMC: peripheral blood mononuclear cell.

**Figure 7:**
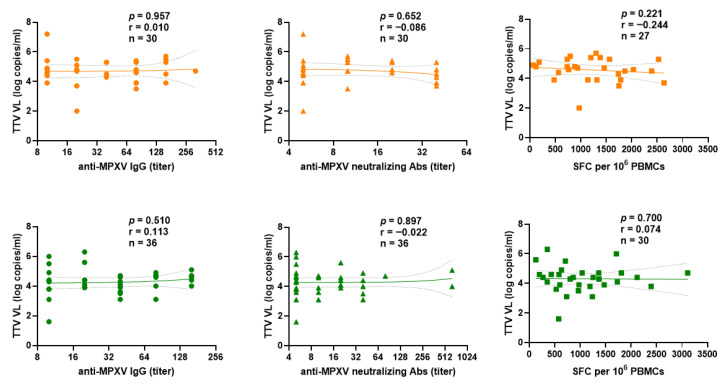
Correlation between pre-vaccination TTV VL and anti-MPXV IgGs (circle), nAbs (triangle) titers and T cell-specific responses to the MVA-BN vaccine (assessed by interferon-γ ELISpot) (square) after second vaccine dose in PLWH (orange) and PLWoH (green) within the Mpox-Vac group. The best-fit line and 95% confidence bands of the linear regression analysis are represented by solid and dotted lines, respectively. Footnotes: TTV: Torquetenovirus, VL: viral load; MPXV: Mpox virus, PBMC: peripheral blood mononuclear cell.

We examined the difference between PLWH and PLWoH within the Mpox-Vac group. The correlation between pre-vaccination TTV VL and responses to the MVA-BN vaccine (anti-MPXV IgGs, nAbs titers, and T cell-specific) after the first (Figure 6) or second (Figure 7) dose showed no significant difference.

## 4. Discussion

In this study, we investigated whether baseline TTV viremia correlates with the magnitude of humoral and cellular immune responses induced by MVA-BN and TAK-003 vaccines in individuals with preserved or only mildly impaired immune function. Although TTV DNA was detectable in the majority of participants, particularly among PLWH, baseline TTV VL did not correlate with vaccine-induced IgG production, neutralizing antibody titers, or antigen-specific T cell responses in most analyses. This is consistent with emerging evidence suggesting that vaccine-induced immunity, particularly against dengue virus, depends on a multifaceted interplay between humoral and cellular responses rather than on single immunological parameters [32,33]. The only statistically significant finding was an inverse correlation between TTV VL and DVG neutralizing antibody titers, which warrants cautious interpretation given the limited sample size, especially in light of recent discussions on vaccine-derived viral variants and their potential impact on immune responses [34]. Importantly, this study was not designed to compare vaccine platforms or populations directly. Instead, the central question addressed was whether baseline TTV viremia predicts vaccine immunogenicity within clinically defined cohorts.

Although negative, these findings highlight several interesting points about TTV association with the immune system. Firstly, they confirmed the concept that TTV viremia reflects the host’s immunological status, and simultaneously, they suggested that the predictive utility of TTV is context-dependent. We know that in strongly immunosuppressed individuals, elevated TTV VLs are consistently associated with reduced immune competence and diminished vaccine responsiveness. On the contrary, as observed in our cohort of immunocompetent or mildly immunosuppressed individuals, the pre-vaccination measure of TTV viremia may not exhibit sufficient fluctuations to capture significant differences in immunological performance. Collectively, our results support a continuum model of TTV utility. In this context, the limited representation of individuals with profound immunosuppression and the lack of more detailed immunological variables may explain the absence of a clearly definable immunosuppression threshold at which TTV acquires strong predictive value for vaccine immunogenicity. The absence of consistent correlations despite marked internal heterogeneity (including HIV status, age, and prior vaccination) further supports the notion that TTV lacks discriminative power in individuals with preserved or mildly impaired immune function. Rather than functioning as a universal predictor of vaccine responsiveness, TTV may serve as a dynamic biomarker whose predictive value emerges only along increasing gradients of immunosuppression. In this framework, TTV VL would not discriminate between individuals within the normal or near-normal immune range but could become clinically informative once immune competence declines beyond a defined threshold. Identifying such thresholds could enable the definition of “immunological risk zones” potentially guiding vaccine scheduling, booster timing, or intensified immunological monitoring in vulnerable populations.

The study provides important information about TTV, but it has some limitations. First, immune markers like immunosenescence CD4+ T cell subsets were unavailable for PLWH, preventing more precise immunological stratification in these patients. Second, the DGV-Vac cohort had a relatively small sample size, limiting statistical power for subgroup analyses and making the observed correlation as exploratory or hypothesis-generating. Third, because the Mpox-Vac and DGV-Vac cohorts represent distinct target populations by design, adjusted inter-group analyses (e.g., propensity score matching) were not performed and would not have supported meaningful causal interpretation. Finally, immune responses in the two cohorts were assessed at different time points relative to vaccination, reflecting protocol-specific schedules rather than a comparative design. In addition, DGV nAbs were measured only against DENV-2 using a live-virus neutralization assay. This choice reflects the well-established robustness and reproducibility of DENV-2 in vitro, as this serotype is the most readily expandable and therefore most commonly used as a functional surrogate in TAK-003 immunogenicity studies. Nevertheless, a full post-vaccination assessment including nAbs against all four DGV serotypes would further strengthen future investigations. Despite these limitations, our study contributes to the growing body of evidence clarifying the clinical boundaries of TTV as an immune biomarker.

## 5. Conclusions

The measurement of TTV viremia is currently unable to reliably predict the magnitude or strength of vaccine-induced immune responses in individuals who are immunocompetent or only mildly immunosuppressed. However, the findings support that TTV may act as a dynamic biomarker of the immunological status of the infected host, with possible predictive value likely emerging along a continuum of immunosuppression. Therefore, further research is needed to clarify this relationship and identify if a specific TTV viremia threshold correlates with clinically relevant immune dysfunction. Establishing such a cut-off could help clinicians interpret TTV levels more accurately in the context of patient immune status. Furthermore, future investigations should also explore the potential utility of TTV viremia as a stratification tool in the design and optimization of vaccine strategies, as well as in broader immunological monitoring.

## Data Availability

The original contributions presented in the study are included in the article; further inquiries can be directed at the corresponding author.
